# Altered Expression of m6A-Associated Genes Is Linked with Poor Prognosis in Pediatric Acute Myeloid Leukemia Patients

**DOI:** 10.3390/biom15091238

**Published:** 2025-08-27

**Authors:** Parminder Kaur, Bollipogu Rajitha, Richa Jain, Pankaj Sharma, Prateek Bhatia, Shano Naseem, Amita Trehan, Minu Singh

**Affiliations:** 1 Hematology Oncology Unit, Department of Pediatrics, Postgraduate Institute of Medical Education and Research, Chandigarh 160012, India; kaur2211parminder@gmail.com (P.K.); rajithabollipogu@gmail.com (B.R.); docrichajain@gmail.com (R.J.); spankaj.1928@gmail.com (P.S.); prateekbhatia16@gmail.com (P.B.); amita911@gmail.com (A.T.); 2 Department of Hematology, Postgraduate Institute of Medical Education and Research, Chandigarh 160012, India; shanonaseem@gmail.com

**Keywords:** acute myeloid leukemia, m6A modification, readers, writers, erasers, clinical correlation

## Abstract

The dysregulation of m6A-related genes recognized as ‘writers’, ‘readers’, and ‘erasers’ is reported to be involved in the initiation, progression, and drug resistance of acute myeloid leukemia (AML). In the present study, we investigated the expression levels of various readers, writers, and erasers in pediatric AML patients. Additionally, we categorized the patients according to the molecular subtyping of common mutations and recurrent fusions and correlated the expression of m6A-associated genes with different molecular subtypes and evaluated their prognostic and clinical implications. A total of fifty-seven patients with pediatric de novo AML were enrolled in the study. The study cohort consisted of 41 males and 16 females with a median age of 7 years (range 1 to 12 years). A high expression of m6A RNA modification complex genes was noted in AML patients. Among the writers, *METTL3* and *METTL14* were found to be upregulated in 19 and 17 patients, the readers *YTHDF1* and *YTHDF2* showed higher expression in 6 and 10 patients, while a high expression of erasers *FTO* and *ALKBH5* was found in 28 patients and 1 patient, respectively. Further, the expression of m6A regulators showed a significant association with genetic alterations including *FLT3-ITD*, *RBM15::MKL* fusions and *NPM1* mutations. Additionally, while evaluating the prognostic implications, both the readers *YTHDF1* and *YTHDF2* showed a significant correlation with TLC at diagnosis (*p* < 0.05). Further, Kaplan–Meier estimation showed a poor event-free survival in cases with the overexpression of *YTHDF1* (log-rank *p* = 0.028). Additionally, we noted a strong correlation between *YTHDF1* overexpression and treatment-related mortality (log-rank *p* < 0.001), and a nearly significant correlation with *YTHDF2* expression in such patients (log-rank *p* = 0.053) at a median follow-up of 8 months. Thus, our data suggest that m6A genes, especially readers *YTHDF1* and *YTHDF2*, are involved in the disease prognosis of AML and probably function in an integrated manner with other m6A-modifying genes to subsequently play a role in AML pathogenesis.

## 1. Introduction

AML is a heterogenous hematological malignancy characterized by the uncontrolled proliferation of myeloid progenitor blast cells. Although most patients initially achieve remission following chemotherapy, 75% of patients relapse and succumb to the disease within 5 years of diagnosis [1]. The pathogenesis and clinical outcome of AML are based on different genetic or epigenetic alterations and/or dysregulations [2,3]. Since DNA mutations are reported to be relatively rare in AML (average of ~13 per patient) compared to other forms of leukemia, regulatory mechanisms are expected to play a crucial role in the development and progression of the disease.

RNA modifications are dynamic and reversible processes that have been shown to play an important regulatory role in cellular homeostasis [4]. There are multiple reports on the development of cancer due to the dysregulation of RNA modifications and their aberrant expression is linked to the initiation, progression, and drug resistance of various cancers, including leukemia [5,6,7,8]. N6-methyladenosine (m6A) is the most prevalent RNA modification found on mRNA. This modification plays an important role in RNA metabolism such as RNA splicing, export, decay, and translation. Sets of proteins recognized as ‘writers’, ‘readers’, and ‘erasers’ are involved in methylation, recognition, and demethylation, respectively [9]. Writers help in achieving methylation and consist of a methyltransferase complex, which contains methyltransferase-like 3 (*METTL3*), methyltransferase-like 14 (*METTL14*), Wilms’ tumor-1-associated protein (*WTAP*), and *HAKAI* (*CBLL1*), among others [10]. Readers recognize methylation to regulate the fate of RNA and consist of YTH domain-containing family protein 1/2/3 (*YTHDF1/2/3*), YTH domain-containing protein 1/2 (*YTHDC1/2*), insulin-like growth factor-binding protein 1/2/3 (*IGF2BP1/2/3*), and heteronuclear ribonucleoproteins (including *HNRNPC*, *HNRNPG*, and *HNRNPA2B1*). Proteins categorized as erasers reverse methylation modification by carrying out a demethylation reaction. The fat mass and obesity-associated protein (*FTO*/*ALKBH9*) and AlkB homolog 5 (*ALKBH5*) are the two demethylases reported so far [11].

A number of studies have demonstrated the dysregulation of m6A-associated genes in AML leukemogenesis. The upregulation of *METTL3* expression is reported in AML cell lines as well as in patients and is associated with chemoresistance by regulating the half-life of *ITGA4* mRNA [6,12]. *METTL3*/*METTL14* are shown to regulate the expression of p53, *CDKN1a/p21*, and mdm2, thus having an oncogenic effect in AML [13]. *WTAP* is differentially expressed in non-M3 AML patients and *FLT3-ITD*-mutated AML patients [14]. Further, the overexpression of *WTAP* in t (8;21) AML patients is reported to be linked with poor prognosis [15]. *YTHDF2* is reported to be significantly upregulated in adult t (8;21) AML patients and is associated with a high risk of relapse [16]. *YTHDF1* is also shown to be upregulated in AML patients and has been demonstrated to promote the translation of cyclin E2, thus aiding in the progression of leukemia [17]. *FTO* is shown to be abnormally regulated in t (11q23)/MLL-rearranged, t (15;17)/*PML::RARA*, *FLT3-ITD*, and/or *NPM1*-mutated AML phenotypes. *FTO* is also demonstrated to enhance its cellular viability and proliferation while inhibiting apoptosis, thus helping in the leukemogenesis of AML [18,19].

As evident by multiple studies, the expression of m6A-associated genes in AML is related to leukemia differentiation and development. While most studies are based on adult AML data, reports from pediatric populations are rare. Further, the expression profiling and clinical correlation of multiple markers of m6A-associated genes in pediatric patient samples have not been reported so far. Our present study investigated the expression levels of various readers, writers, and erasers in pediatric AML patients. Additionally, we categorized the patients via the molecular subtyping of common mutations such as *NPM1* and *FLT3*, as well as the recurrent fusions, including *AML-1::ETO*, *CBFB::MYH11*, *PML::RARA*, etc. We correlated the m6A-associated gene expression with different molecular subtypes and evaluated their prognostic and clinical implications. Together, the data demonstrated important roles of *YTHDF1/2* and *METTL3/14* in prognosis and survival of AML patients.

## 2. Methodology

### 2.1. Study Population

Fifty-seven newly diagnosed pediatric AML patients (age ≤ 12 years) and twenty-three age-matched healthy controls were enrolled in the study. Cases were confirmed based on morphology, immunophenotyping, and cytogenetic analysis. Patients were treated with standard chemotherapy followed at our institute based on a modified UK MRC 15 protocol with cytarabine, daunorubicin, and etoposide (ADE) for 2 courses followed by high-dose cytarabine for 2 courses [20]. Patients were followed up for post-induction bone marrow to assess morphological remission (<5% blast in marrow). Minimal residual disease assessment was available only in selected patients in the study period, hence it was not used for assessing response to therapy in this study. The outcome of the patients was evaluated based on events including relapse, progressive disease, or death. Written informed consent in agreement with the Declaration of Helsinki was obtained from the patients for participation in the study. The study was approved by the Institutional Ethics Committee, PGIMER.

### 2.2. DNA and RNA Isolation

Peripheral blood mononuclear cells (PBMCs) were isolated from the patient’s blood samples when the patient was diagnosed (before starting the therapy) followed by DNA and RNA isolation using a DNA and RNA blood kit (Qiagen Co., Ltd., Hilden, Germany), respectively, as per the manufacturer’s protocol. RNA and DNA were quantified using NanoQuant (Tecan Inc, Tecan, Zürich, Switzerland). cDNA synthesis from RNA was performed using the first strand cDNA synthesis kit (Thermo Fisher Scientific, New York, NY, USA).

### 2.3. Molecular Subtyping

Molecular subtyping was performed using the TRUPCR Acute Leukemia Panel Kit (3B Black Bio India Ltd., Bhopal, India) according to the manufacturer’s protocol. The presence of *AML1::ETO*, *CBFB::MYH11*, *PML::RARA*, *DEK::NUP214*, *SET::NUP214*, *BCR::ABL1*, *RBM15-MKL1*, and *C-KIT* mutation was investigated with cDNA using specific a primer–probe mix using RT-qPCR (QuantStudio 5, Applied Biosystems, TFS, Singapore). The sample Ct values were analyzed with internal control ABL1. The presence of *C-KIT* and *NPM1* (Types A, B, and D) was investigated with DNA using qPCR. The sample was considered positive if the ΔCt between mutation-specific amplification and reference control was within the defined cut-off values as per manufacturer guidelines. *FLT3-ITD* mutation was detected using conventional PCR followed by gel electrophoresis. The sample was considered positive if there is the presence of an additional band larger than wild type 330 bp product. Positive and negative controls were run for all the experiments.

### 2.4. Expression Level Measurement of m6A-Associated Genes

cDNAs from the patient samples and age-matched healthy controls were subjected to RT-qPCR by using PowerUP SYBR green PCR master mix (Thermo Fisher Scientific, Vilnius, Lithuania) using a QuantStudio 5 Real-Time PCR System (QuantStudio 5, Applied Biosystems, TFS, Singapore). The expression profiles of *METTL3*, *METTL14*, *WTAP*, *YTHDF1*, *YTHDF2*, *FTO*, and *ALKBH5* were analyzed. Primers used are listed in Table S1. *GAPDH* was used as an internal control. All the samples were run in triplicates. ΔCt was calculated by normalizing with endogenous control Ct values. ΔΔCt was calculated by subtracting the ΔCt of control samples and fold change in expression was calculated by 2^−ΔΔCt^ [21]. Fold change (2^−ΔΔCt^) of ≥2 was taken as positive for high expression in the patient sample compared to the expression level of age-matched healthy controls. This threshold indicates that the gene expression in patient samples was at least two times higher than the baseline expression observed in controls.

### 2.5. Statistical Analysis

Continuous variables were expressed as mean/median with range or interquartile range (IQR) for follow-up and categorical variables as ratio/proportion. Chi-square and Fisher exact tests were performed to check the association between the categorical variables. Survival curves were calculated using the Kaplan–Meier curve and log-rank tests. Spearman R correlation was performed to check the correlation between expression and subtypes. The data analysis was carried out using SPSS v25.0 and GraphPad prism 8.0.2 software. A *p* value of <0.05 was considered significant.

## 3. Results

### 3.1. Clinical Characteristics of Pediatric AML Patients at Diagnosis

A total of fifty-seven patients with de novo AML were enrolled in the study. The study cohort consisted of 41 males and 16 females with a median age of 7 years (range 1 to 12 years). The median WBC count at diagnosis was 33,800/µL (range 300–690,000/µL). Out of the 47 patients, 11 had a blast count of ≤50% (range 23–50%) while 36 patients had a blast count of >50% (range 52–98%). Based on morphology, 3 patients were classified as subtype M0, 12 as M1 and M2 each, 3 as M3, 12 as M4, 7 as M5, and 3 patients as M7 subtype. Five patients remained unclassified. Out of 46 patients, 36 patients achieved morphological remission post-course 1 of induction therapy while 10 were given additional induction therapy to achieve remission. Further, six patients did not achieve complete remission at the end of induction therapy. The median follow-up was 8 months (IQR: 14 months). The details of various clinical and hematological parameters are listed in Table 1.

### 3.2. Expression Profile of m6A-Associated Genes in Pediatric AML Patients

**Figure 1:**
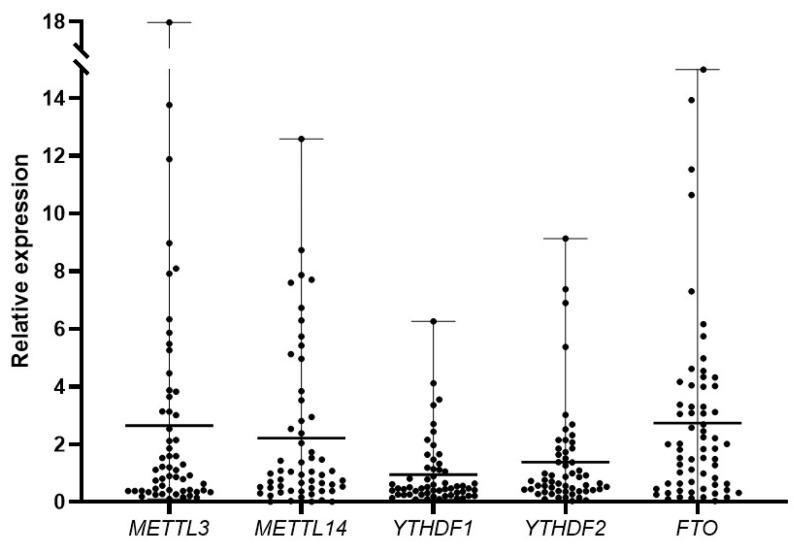
Relative fold change in mRNA levels of m6A regulators in pediatric AML patients as compared to controls (n = 57). Values are plotted as median with range.

Among writers of the RNA modification complex, *METTL3*, *METTL14*, and *WTAP* were analyzed for their expression levels in pediatric AML patients. In our cohort, *METTL3* was found to be upregulated in 19 patients. The fold change ranged from 2 to 18 compared to the control group. *METTL14* was upregulated in 17 patients with the fold change ranging from 2 to 12.5. However, the expression of *WTAP* was not found to be elevated in pediatric AML patients and was similar to that of the control group. From the *YTH* family of m6A reader proteins, *YTHDF1* and *YTHDF2* were selected for examining their expression levels. Six patients showed higher expression of *YTHDF1* as compared to the controls and the fold change ranged from 2 to 6.3. The expression of *YTHDF2* was found to be higher in 10 patients with fold change ranging from 2 to 9. The relative expressions of both the demethylases *FTO* and *ALKBH5* were checked in pediatric AML patients. The expression level of *FTO* was higher in 28 patients with fold change ranging from 2 to 15. *ALKBH5* was upregulated only in one patient with a relative fold change of 2.7. Thus, *FTO* was noted to have high expression in the most patients (n = 28) among all m6A modifying genes studied, followed by *METTL3* (n = 19) and *METTL14* (n = 17). Further, the level of expression was highest for *METTL3* (maximum fold change 18) followed by *FTO* (maximum fold change 15) and *METTL14* (maximum fold change 12.5). The relative expression of different m6A markers showing high expression patterns is depicted in Figure 1 as a dot plot.

### 3.3. Correlation of Expression Profiles of m6A-Related Genes with Various Clinicopathological Factors

The expression of *METTL3*, *METTL14*, *YTHDF1*, *YTHDF2*, *FTO*, and *ALKBH5* was correlated with various clinical parameters such as age, gender, TLC at diagnosis, initial blast count, remission post-induction (following course 1 and with an additional course, if any), and events such as death, relapse, and progressive disease. Though high expression of m6A genes was noted in a significant number of cases, their correlation with clinical factors could not reach the level of significance in most of the cases. However, we did find the expression of both the readers analyzed, i.e., *YTHDF1* and *YTHDF2*, to have a significant correlation with TLC at diagnosis (*p* < 0.05). Low expression of *YTHDF1* and *YTHDF2* is associated with low TLC. The correlation of expression profiles of m6A-related genes with various clinical factors is listed in Table 2.

### 3.4. Molecular Subtyping of Pediatric AML Patients for Common Mutations and Fusions

**Figure 2:**
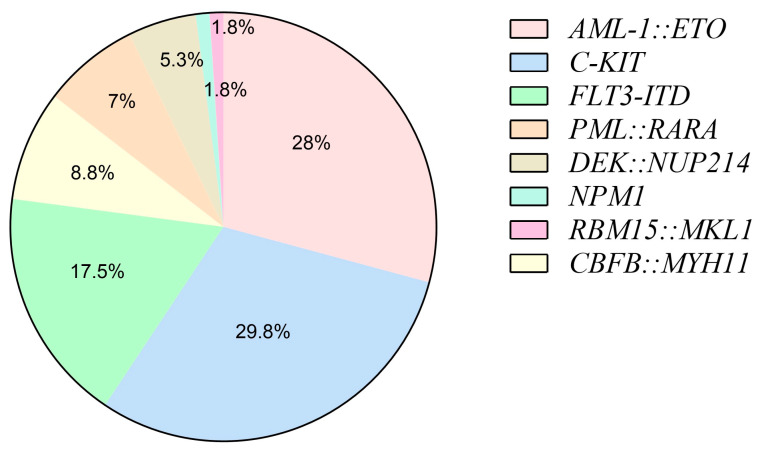
Frequency of cases (in percent) across various gene fusions and mutations tested in pediatric AML patients (n = 57).

Further, we wanted to see if the expression of m6A-associated genes differs in the various molecular subtypes known and is related to disease prognosis in AML. Therefore, we performed the molecular subtyping of the patients and correlated the expression of m6A-associated genes among different subtypes. Out of 57 patients, 39 patients showed at least one of the fusion or mutation tested while 18 patients did not show any common abnormality tested in the study. The frequency of *C-KIT* mutation was noted to be highest, with 30% (n = 17) of the patients harboring it, followed by *AML-1::ETO* gene fusion in 28% (n = 16) of patients which results from translocation t (8;21) (q22;q22). Seven patients having t (8;21) showed the presence of *C-KIT* mutation. *FLT3-ITD* mutation was noted in 18% (n = 10) of patients. Nine percent (n = 5) of patients showed the presence of *CBFB::MYH11* fusion resulting from inv (16) (p13;q22) while seven percent (n = 4) of the patients showed the presence of *PML::RARA* chimeric transcript resulting from the translocation t (15;17) (q22;q12). Five percent (n = 3) of the patients showed the presence of the *DEK-CAN* gene which results from the t (6;9) (6p22.3;9q34.1) while both the *NPM1* mutation and *RBM15::MKL1* fusion gene were noted to be present in only two percent (n = 1) of the total patients. None of the patients showed the presence of *SET::CAN* or *BCR::ABL1* gene fusion in the study cohort. The distribution of molecular subtypes in AML patients has been shown as a pie chart in Figure 2.

### 3.5. Differential Expression of m6A-Associated Genes in Different Subtypes in Pediatric AML Patients

**Figure 3:**
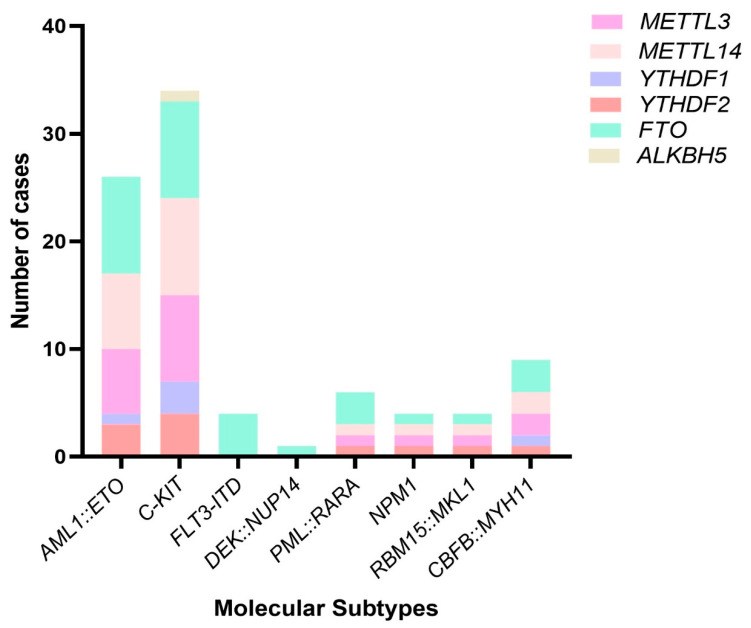
Case distribution on the basis of m6A-associated gene overexpression, across different molecular subtypes in AML patients.

To examine the correlation of m6A genes with common fusions and mutations in AML, we analyzed the expression data with molecular subtypes of patients. The distribution of cases showing high expression of m6A genes among categories of molecular subtypes is depicted in Figure 3.

**Figure 4:**
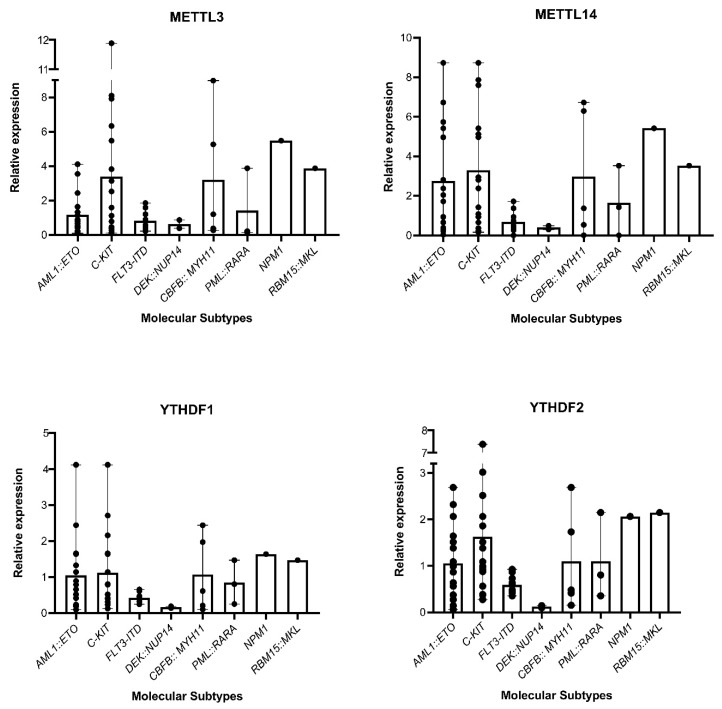
Presence of different fusions and mutations commonly present in AML in cases with overexpression of *METTL3/14* and *YTHDF1/2*.

We noted that the presence of *FLT3-ITD* mutation had a significant correlation with the expression levels of *METTL3* and *METTL14* (*p* < 0.05). Additionally, *METTL14* expression was significantly associated with the presence of *C-KIT* mutations (*p* < 0.05). Furthermore, *YTHDF2* expression was significantly correlated with the presence of *RBM15::MKL1* and *NPM1* mutations (*p* < 0.005). The expression pattern of *METTL3/14* and *YTHDF1/2* across different molecular subtypes is shown in Figure 4 and the correlation is detailed in Table 3.

**Figure 5:**
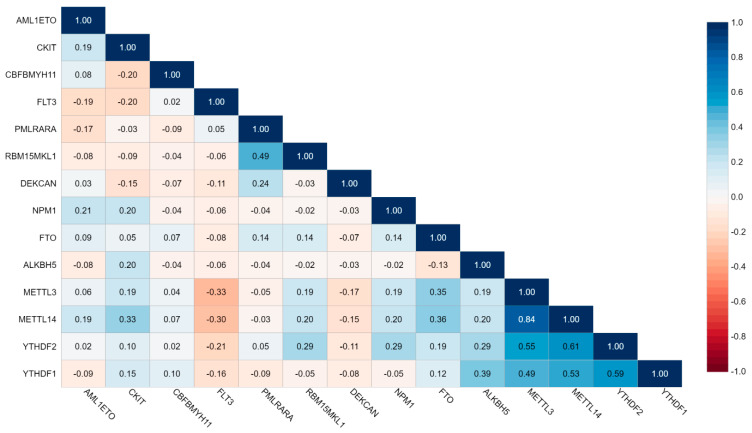
Correlation matrix of molecular subtypes and m6A gene expression in pediatric AML patients (n = 57). The gradient of blue and red colors represents positive and negative correlation, respectively, based on the Spearman correlation rank score.

In order to check the strength and type of correlation among various molecular subtypes in AML patients as well as with m6A-associated genes, Spearman rank correlation was performed. The presence of *PML::RARA* fusion was found to be negatively correlated with *AML1::ETO* (Spearman r = −0.17, *p* = 0.049). Also, the presence of *CBFB::MYH11* was negatively correlated with the presence of *C-KIT* mutations (Spearman r = −0.20, *p* = 0.026). Further, while examining the correlation of *METTL3*, *METTL14*, *YTHDF1*, *YTHDF2*, *FTO*, and *ALKBH5* with molecular subtypes, the presence of *FLT3-ITD* mutations was noted to be negatively correlated with expression of *METTL3* (Spearman r = −0.33, *p* = 0.001), *METTL14* (Spearman r = −0.30, *p* = 0.002), *YTHDF1* (Spearman r = −0.16, *p* = 0.029), and *YTHDF2* (Spearman r = −0.21, *p* = 0.008). Additionally, the expression of *METTL14* was positively correlated with the presence of *C-KIT* (Spearman r = 0.33, *p* = 0.025). We also noted that the expressions of various m6A-associated genes are correlated with each other in AML patients. *METTL3* expression was positively correlated with *METTL14* (Spearman r = 0.84, *p* < 0.001), *YTHDF1* (Spearman r = 0.49, *p*= 0.007), *YTHDF2* (Spearman r = 0.55, *p* < 0.001), and with *FTO* (Spearman r = 0.35, *p* = 0.008). Additionally, the expressions of *METTL14* and *FTO* (Spearman r = 0.36, *p*= 0.006) and the expressions of *YTHDF1* and *YTHDF2* were also found to be positively correlated with each other (Spearman r = 0.59, *p* = 0.017). The details of correlation analysis with Spearman rank scores are provided in Figure 5.

### 3.6. Correlation of m6A-Related Genes with Clinical Outcome in AML Patients

**Figure 6:**
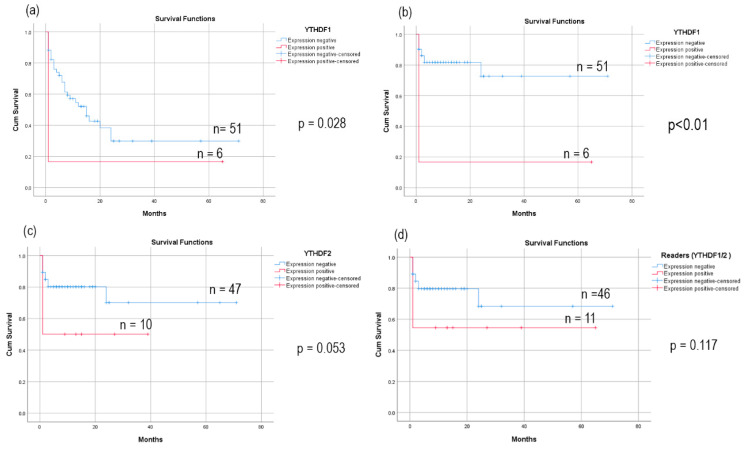
Kaplan–Meier survival curves of cases showing overexpression of m6A readers for poor event-free survival with *YTHDF1* (**a**) and for treatment-related mortality with *YTHDF1* (**b**), *YTHDF2* (**c**) and for both the readers *YTHDF1* and 2 (**d**) in pediatric AML patients (n = 57) with a median follow-up of 8 months (IQR: 14 months).

Further, to check the prognostic implications of m6A-associated genes, the high- and low-expression cases were analyzed for relapse and survival by Kaplan–Meier estimation (Figure 6). The median period of follow-up was 8 months (IQR: 14 months) and the data were censored when the patient died, relapsed, or at last follow-up for progressive disease. The cases positive for *YTHDF1* overexpression showed poor event-free survival (log-rank *p*= 0.028) as shown in Figure 6a. Further, we noted that cases with early deaths during treatment probably due to treatment-related toxicities had a significant correlation with overexpression of *YTHDF1* (log-rank *p* < 0.001, Figure 6b). Additionally, we noted that the cases positive for *YTHDF2* expression also had a nearly significant correlation with treatment-related mortalities (log-rank *p* = 0.053) and a trend of poor survival in cases expressing either *YTHDF1* or *YTHDF2* (Figure 6b,c). Thus, the data support the prognostic implications of both the readers *YTHDF1* and *YTHDF2* in the survival of pediatric AML patients. However, we did not find any correlation between the expression of other m6A-associated genes under investigation and with relapse or survival of the patients.

## 4. Discussion

AML is a heterogeneous disease having a higher mortality rate as compared to other leukemias in children like acute lymphoblastic leukemia (ALL). Due to the low rate of DNA mutations compared to other forms of leukemia, AML presents a special challenge in developing new targets of therapy. This is evident by the fact that treatment options for AML have been limited for decades while great advancements have been made in therapies for most blood cancer types [22]. Exploring different pathways to develop novel therapies is an ongoing quest in AML therapy research. Recently described m6A-associated modifications have shown promising data on their involvement in AML pathogenesis and drug development [23]. However, the reports are restricted to either in silico data analysis or adult AML patient studies. In the present study, we focused on analyzing the expression profile of various m6A-associated genes in pediatric AML patients in order to investigate their role in disease prognosis. We also checked the frequency and occurrence of common genetic mutations and fusions in AML and examined whether the m6A genes are in any way associated with them.

Our cohort of pediatric patients had a median age of 7 years with a predominance of male patients which is consistent with the literature [24,25]. We checked the expression of different m6A genes in our cohort and found that the expression of *METTL3*, *METTL14*, *YTHDF1*, *YTHDF2*, and *FTO* was upregulated in our cohort as compared to the control group. m6A complex writers *METTL3*/*METTL14* are shown to be overexpressed in AML in various studies and play an important role in myeloid development of cells [25,26]. Further, overexpression of *METTL3* is shown to be associated with adverse treatment outcomes and chemoresistance in relapsed AML patients younger than 60 years [6]. Another study on 89 patients consisting majorly of adult patients has reported that *METTL3*/*METTL14* are highly expressed in AML with shorter survival in the patients and play an oncogenic role in AML by targeting the mdm2/p53 signaling pathway [13]. Our study is in coherence with the literature supporting the involvement of *METTL3*/*METTL14* in AML pathogenesis. We also noted a positive correlation in the expression of *METTL3* and *METTL14* in our cases. This could probably be the reason that *METTL14* forms the dimer with *METTL3* for the catalytic activity of *METTL3* [27].

*YTHDF1* is the most abundant reader protein which plays an important role in RNA stability while *YTHDF2* plays a role in RNA decay. They are shown to be upregulated in various cancers such as breast cancer, gastric cancer, esophageal cancer, colorectal cancer, liver cancer, prostate and bladder cancer, etc. [28,29,30,31]. *YTHDF1* and *YTHDF2* were found to be overexpressed in pediatric AML patients of our cohort. Paris et al. have reported recently that *YTHDF2* is highly expressed across human AML compared to normal human CD34^+^ cells and is essential for leukemia initiation. Further, in a study by Chen Zi et al. in adult AML patients, *YTHDF2* mRNA expression is shown to be elevated in t (8;21) AML patients and is associated with a higher risk of relapse and inferior relapse-free survival [16]. A study by Zhu Y et al. has shown that high expression of *YTHDF1* contributes to the progression and metastasis of cancer by regulating RNA stability or promoting translation [28]. Further, aberrant expression of *YTHDF2* in AML patients, especially in relapsed cases, has been shown to play an oncogenic role in AML [29]. We noted that *YTHDF1* has a significant correlation to TLC count at diagnosis as well as with survival in AML children during treatment. Most of the patients having low YTHDF1 expression showed low TLC. Kaplan–Meier analysis also showed that higher expression of *YTHDF1* is linked with poor event-free survival in AML patients. Interestingly, we also noted that overexpression of *YTHDF1* and *YTHDF2* is linked with deaths during the early phase of treatment, probably due to toxicity. We tried to analyze the relationship between treatment drugs and the expression patterns of *YTHDF1/2* readers but did not find any significant results. However, it is worth noting that four out of five cases positive for *YTHDF1* expression had died or succumbed to death during or post-course 1 of therapy. Although, due to the limited case numbers, this analysis did not reach the level of significance, these data cannot be ignored and need further investigation and validation in a larger cohort in the future. In addition, our data support the previous observation and corroborate the important role of *YTHDF1* and *YTHDF2* in the disease pathogenesis of AML, which holds true in the pediatric population as well.

One of the most interesting genes involved in m6A modification is *FTO*. Since the discovery of its role as an eraser, *FTO* has been the focus of research on various cancers, especially drug development. In AML, studies have shown a high level of *FTO* expression in patients [19,30]. We also noted high expression of *FTO* in our patients ranging from 2- to 12-fold change. Further, it has been reported as a critical oncogenic molecule that enhances leukemic oncogene-mediated cell transformation and leukemogenesis [19]. *FTO* has also been shown to promote leukemic cell survival in facilitating the cell cycle and inhibiting cell apoptosis in adult *NPM1*-mutated AML [31]. Additionally, Li et al. have shown that *FTO* enhances leukemic oncogene-mediated cell transformation and leukemogenesis and inhibits all-trans-retinoic acid (ATRA)-induced AML cell differentiation. In their study, they also reported that *ALKBH5*, which is another eraser identified recently, did not have elevated expression in AML. We noted similar observations in the case of *ALKBH5* in our cohort of AML patients. Out of fifty-seven cases enrolled, only one case showed high expression of *ALKBH5* while we observed high expression of *FTO* in 49% of pediatric AML patients. Though the number of cases showing elevated *FTO* expression was high, *FTO* could not be correlated with clinical factors leading to prognostic importance in our cohort. Studies have shown that elevated expression of *FTO* occurs in a specific subset of cases such as with t (11q23)/MLL rearrangements, t (15;17)/*PML::RARA*, *FLT3-ITD* and/or *NPM1* mutations [19], and t (8;21) AML [32]. Therefore, its role in disease pathogenesis could be limited to the said molecular subtypes. Nonetheless, it is important to note that there are contradicting reports in the literature in relation to the expression of *FTO* and its role in AML pathogenesis. For example, Zhou W et al. in their study have reported that FTO expression is upregulated in the *AML1::ETO* subtype of AML in adult patients and have demonstrated that *AML1::ETO* upregulates *FTO* mechanistically, through inhibiting the transcriptional repression of *FTO* mediated by transcription factor PU.1. In addition, through a positive regulatory loop, *FTO* promotes the expression of *AML1::ETO* by inhibiting *YTHDF2*-mediated *AML1::ETO* mRNA decay [33]. Meanwhile, in our study based on pediatric cases, we did not find any significant difference in cases with elevated *FTO* expression among *AML1::ETO* positive and negative cases. A report by Li Z et al. corroborates our data by demonstrating that *FTO* is elevated in MLL-rearranged AML and, among non-MLL-rearranged AMLs, *FTO* is expressed at a significantly higher level in t (15;17) AML and not in t (8;21), i.e., *AML1-ETO* and inv (16) AMLs [19]. Thus, considering the contraindicative studies in the literature, it is suggested that a larger cohort of pediatric AML patients should be evaluated before making any conclusion regarding the role of *FTO* as an oncogene in pediatric AML.

Further, upon examining the correlation of expression of m6A modifiers with common genetic alterations in AML, interestingly, we noted that *FLT3-ITD* mutation was negatively correlated with the expression of *METTL3*, *METTL14*, *YTHDF1*, and *YTHDF2*. The presence of *FLT3-ITD* mutations is linked to poor prognosis in AML patients and there are ongoing studies on the usage of *FLT3* inhibitors as a treatment option for AML patients. Studies detailing the composite role of common genetic subtypes with m6A genes are rare. Li Z. et al. have reported that AML patients with normal karyotypes have high expression of *FTO* in cases positive for *FLT3-ITD* and/or *NPM1* mutations [19]. In our study population, *FLT3-ITD* positive cases had only *FTO* as a high-expression protein gene among m6A genes except for one case showing high expression of *ALKBH5* as shown in Figure 3. Thus, our observation is in coherence with the previous study suggesting that expression of *FTO* could be involved in worse prognosis of *FLT-ITD* mutation-positive AML cases. An in-depth study investigating their relationship and molecular mechanism is needed to unfold their role that will help in identifying novel targets of therapy. There are various reports which show that targeting the m6A modifiers with inhibitors can result in a decrease in cancer progression [34,35,36]. Treatment of mouse AML cells and MOLM13 cells with STM2457 inhibitor has been shown to decrease AML cell progression [37]. Analogues of meclofenamic cells FB23 and FB23-2 which inhibit *FTO* expression are also shown to have an inhibitory effect on AML cell lines [38]. These reports are limited to in vivo studies only and using them for therapeutics requires more detailed validation studies.

## 5. Conclusions

M6A regulators are important key players in AML pathogenesis, and our data provide valuable input from pediatric AML patients. Expression profiling of m6A genes is an imperative base for further studies on identifying pathways of regulation and molecular mechanisms for their role in cancer progression. Our data validated on patients are a significant contribution to m6A pathway research. Future studies on larger cohorts and functional validation will provide factual data that could be utilized in novel therapeutic interventions that are the need of the hour in AML management.

## Figures and Tables

**Table 1:** Clinical and hematological characteristics of pediatric AML patients enrolled for the study (n = 57).

Clinical and Hematological Parameters	AML	
Age (n = 57)	Range 1–12 years (≤5 = 33, ≥5 = 34)	Median = 7 years
Gender (n = 57)	Male = 41; Female = 16	Ratio (M:F) = 2.6:1
WBC count (×10^3^/µL) (n = 55)	Range 300–690,000	Median = 33,800
Remission post-induction course 1 (n = 46)	Positive: 36	Negative: 10
Remission post-induction (n = 46)	Positive: 40	Negative: 06
Percent blast count at diagnosis (n = 47)	≤50% = 11 ≥50% = 36	
FAB subtype (n = 52)	M0 = 3	
	M1 = 12	
	M2 = 12	
	M3 = 03	
	M4 = 12	
	M5 = 07	
	M7 = 03	
Events (n = 57)	Death = 15	
	Relapse = 15	
	Progressive disease = 06	

n = total number of cases.

**Table 2:** Correlation of m6A-associated genes with various clinical and hematological parameters in pediatric AML patients (n = 57). Relative fold change in expression of more than 2-fold is taken as positive while less than 2-fold is taken as negative. *p* values <0.05 are taken as significant and are represented in bold.

	*METTL3*		*p* Value	*METTL14*		*p* Value	*YTHDF1*		*p* Value	*YTHDF2*		*p* Value	*FTO*		*p* Value	*ALKBH5*		*p* Value
	+	−		+	−		+	−		+	−		+	−		+	−	
**Age (in years, n = 57)**																		
≤5	8	15	0.849	7	16	0.934	3	20	0.611	4	19	0.98	9	14	0.215	1	22	0.22
>5	11	23		10	24		3	31		6	28		19	15		0	34	
**Gender (Male = M, Female =F, n = 57)**																		
M	13	28	0.677	13	28	0.619	4	37	0.762	7	34	0.881	18	23	0.207	1	40	0.529
F	6	10		4	12		2	14		3	13		10	6		0	16	
**TLC (×10^3^/µL, n = 55)**																		
<10	13	34	0.052	12	35	0.159	3	44	**0.009**	6	41	**0.012**	23	24	0.549	0	47	**0.014**
≥10	5	3		4	4		3	5		4	4		3	5		1	7	
**Remission post-course 1 (n = 46)**																		
Yes	11	25	0.973	10	26	0.62	1	35	0.594	4	32	0.92	19	17	0.976	0	36	-
No	3	7		2	8		0	10		1	9		5	5		0	10	
**Remission post-induction (n = 46)**																		
Yes	12	28	0.869	11	29	0.573	1	39	0.695	5	35	0.359	21	19	0.909	0	40	-
No	2	4		1	5		0	6		0	6		3	3		0	6	
**Percent blast count at diagnosis (in %, n = 47)**																		
≤50	3	8	0.588	4	7	0.718	0	11	0.147	0	11	0.065	6	5	0.918	0	11	0.576
>50	13	23		11	25		6	30		9	27		19	17		1	35	
**Subtypes (n = 52)**																		
M0	1	2	0.602	3	0	0.634	0	3	0.932	0	3	0.788	3	0	0.053	0	3	0.364
M1	4	8		9	3		1	11		3	9		5	7		0	12	
M2	6	6		7	5		2	10		2	10		9	3		0	12	
M3	1	2		2	1		0	3		1	2		2	1		0	3	
M4	3	9		9	3		1	11		1	11		2	10		0	12	
M5	1	6		6	1		1	6		1	6		3	4		1	6	
M6	0	0		0	0		0	0		0	0		-	-		0	0	
M7	0	3		3	0		0	3		0	3		1	2		0	3	
**Events**																		
**Relapse (n = 57)**																		
Yes	3	12	0.202	4	11	0.755	0	15	0.122	1	14	0.197	8	7	0.704	0	15	0.547
No	16	26		13	29		6	36		9	33		20	22		1	41	
**Death (n = 57)**																		
Yes	5	10	1	5	10	0.729	5	10	0.001	5	10	0.061	7	8	0.825	1	14	0.091
No	14	28		12	30		1	41		5	37		21	21		0	42	
**Progressive disease (n = 57)**																		
Yes	2	4	1	1	5	0.456	0	6	0.374	0	6	0.232	3	3	0.964	0	6	0.621
No	17	34		16	35		6	45		10	41		25	26		2	49	

n = total number of cases, (+) = positive, (−) = negative.

**Table 3:** Correlation of m6A-associated genes expression with molecular subtypes in pediatric AML patients (n = 57). Relative fold change in expression of more than 2-fold is taken as positive while less than 2-fold is taken as negative. *p* value < 0.05 is taken as significant and are represented in bold.

	*METTL3*		*p* Value	*METTL14*		*p* Value	*YTHDF1*		*p* Value	*YTHDF2*		*p* Value	*FTO*		*p* Value	*ALKBH5*		*p* Value
	+	−		+	−		+	−		+	−		+	−		+	−	
***AML1-ETO* (n = 57)**																		
+	6	10	0.677	7	9	0.151	1	15	0.511	3	13	0.881	9	7	0.501	0	16	0.529
−	13	28		10	31		5	36		7	34		19	22		1	40	
***CBFB-MYH11* (n = 57)**																		
+	2	3	0.741	2	3	0.603	1	4	0.47	1	4	0.88	3	2	0.61	0	5	0.754
−	17	35		15	37		5	47		9	43		25	27		1	51	
***RBM15 MKL1* (n = 57)**																		
+	1	0	0.154	1	0	0.122	0	1	0.729	1	0	**0.029**	1	0	0.305	0	1	0.893
−	18	38		16	40		6	50		9	47		27	29		1	55	
***NPM1* (n = 57)**																		
+	1	0	0.154	1	0	0.122	0	1	0.729	1	0	**0.029**	1	0	0.305	0	1	0.893
−	18	38		16	40		6	50		9	47		27	29		1	55	
***C-KIT* (n = 57)**																		
+	8	9	0.152	9	8	**0.013**	3	14	0.253	4	13	0.439	9	8	0.707	1	16	0.122
−	11	29		8	32		3	37		6	34		19	21		0	40	
***DEK-CAN* (n = 57)**																		
+	0	3	0.208	0	3	0.246	0	3	0.542	0	3	0.412	1	2	0.52	0	3	0.812
−	19	35		17	37		6	48		10	44		27	27		1	53	
***FLT3-ITD*** **(n = 57)**																		
+	0	10	**0.014**	0	10	**0.023**	0	10	0.232	0	10	0.108	4	6	0.525	0	10	0.642
−	19	28		17	30		6	41		10	37		24	23		1	46	
***PML-RARA* (n = 57)**																		
+	1	3	0.714	1	3	0.827	0	4	0.477	1	3	0.684	3	1	0.283	0	4	0.782
−	18	35		16	37		6	47		9	44		25	28		1	52	

(+) = expression positive, (−) = expression negative.

## Data Availability

All the data have been included in the manuscript.

## Supplementary Materials

The following supporting information can be downloaded at: https://www.mdpi.com/article/10.3390/biom15091238/s1.

## Abbreviations

ALL, Acute Lymphoblastic Leukemia; AML, Acute Myeloid Leukemia; AML-1::ETO, Acute Myeloid Leukemia 1::Eight-Twenty-One; ATRA, All-Trans Retinoic Acid; BCR::ABL1, Breakpoint Cluster Region::Abelson Murine Leukemia Viral Oncogene 1; CBFB::MYH11, Core-Binding Factor; Beta::Myosin Heavy Chain 11; CDKN1a/p21, Cyclin Dependent Kinase Inhibitor 1A/p21; C-KIT, Cluster of Differentiation-KIT; FLT3-ITD, FMS-like Tyrosine Kinase 3-Internal Tandem Duplication; FTO, Fat Mass and Obesity-Associated protein; GAPDH, Glyceraldehyde-3-Phosphate Dehydrogenase; HAKAI, E3 Ubiquitin-Protein Ligase Hakai; HNRNPA2B1, Heterogeneous Nuclear Ribonucleoprotein A2/B1; HNRNPC, Heterogeneous Nuclear Ribonucleoprotein C; HNRNPG, Heterogeneous Nuclear Ribonucleoprotein G; IGF2BP1/2/3, Insulin-like growth factor-binding protein 1/2/3; ITGA4, Integrin Subunit Alpha 4; M6A, 6-methyladenosine; mdm2, Mouse Double Minute 2 Homolog; METTL14, methyltransferase-like 14; METTL3 methyltransferase-like 3; MLL-rearrangements, Mixed Lineage Leukemia (MLL) Gene Rearrangements; NPM1, Nucleophosmin 1; PBMC, Peripheral blood mononuclear cells; PML::RARA, Promyelocytic Leukemia::Retinoic Acid Receptor Alpha; PU.1, Purine-rich Box 1; RBM15::MKL1, RNA Binding Motif Protein 15::Megakaryoblastic Leukemia 1; SET::NUP214, SET Nuclear Proto-Oncogene::Nucleoporin 214; TLC, Total Leukocyte Count; WBC, White Blood Cell; WTAP, Wilms’ tumor-1 associated protein; YTHDC1/2, YTH domain-containing protein ½; YTHDF1/2/3, YTH domain-containing family protein 1/2/3.
