# Supplementary material for: Altered Expression of m6A-Associated Genes Is Linked with Poor Prognosis in Pediatric Acute Myeloid Leukemia Patients
Source: Biomolecules. 2025 Aug 27;15(9):1238. doi: 10.3390/biom15091238 (PMC12467299; doi:10.3390/biom15091238)
Supplement: Supplementary file 1 [file biomolecules-15-01238-s001.zip › biomolecules-3584802-supplementary.pdf]

Supplementary table: List of RT-PCR primers used in this study.

METTL3	Forward	5’-ATGGGAAGGAACACTGCTTG-3’
	Reverse	5’- ATGACTGGTGGAACGAACCT -3’
METTL14	Forward	5'-ATCGCCTCCTCCCAAATCTA-3'
	Reverse	5'-ACCTCTGTGTGCTCCTCCAC-3'
WTAP	Forward	5’-CTTCCCAAGAAGGTTCGATTGA-3'
	Reverse	5'-TCAGACTCTCTTAGGCCAGTTAC-3’
YTHDF2	Forward	5' CCTTAGGTGGAGCCATGATTG 3'
	Reverse	5' TCTGTGCTACCCAACTTCAGT 3'
YTHDF1	Forward	5' TCAGGCTGGAGAATAACGA 3'
	Reverse	5' GGTTGTGTGCTTGTAGGAACT 3'
ALKBH5	Forward	5’-CGGCGAAGGCTACACTTACG-3’
	Reverse	5’-CCACCAGCTTTTGGATCACCA-3’
FTO	Forward	5’-ACTTGGCTCCCTTATCTGACC-3’
	Reverse	5’-TGTGCAGTGTGAGAAAGGCTT-3’
GAPDH	Forward	5’-GTCTCCTCTGACTTCAACAGCG-3’
	Reverse	5’-ACCACCCTGTTGCTGTAGCCAA-3’

ΔCt Values of Patients and controls obtained from RT-qPCR analysis

	METTL3		METTL14		WTAP		YTHDF1		YTHDF2		FTO		ALKBH5	
S.No.	Patient	Control	Patient	Control	Patient	Control	Patient	Control	Patient	Control	Patient	Control	Pateint	Control
1	4.33	4.54	4.76	4.88	2.91	3.17	4.71	4.11	2.20	3.75	2.03	3.19	3.54	2.03
2	3.61	5.31	4.21	6.49	2.10	2.77	3.72	4.56	2.41	3.37	2.39	1.87	3.38	2.41
3	2.16	4.92	2.31	5.11	3.58	3.05	2.05	3.83	0.88	3.24	2.31	4.95	2.89	4.49
4	4.04	4.28	4.91	4.27	3.53	2.64	4.05	3.56	2.59	2.84	2.60	2.17	3.51	2.34
5	4.92	7.20	5.85	6.68	7.12	4.22	6.73	5.04	6.64	5.20	3.46	3.63	2.96	5.67
6	7.50	5.77	4.31	5.67	7.33	3.40	4.19	4.03	3.42	3.99	4.68	0.91	4.78	3.79
7	3.63	5.28	4.27	4.90	4.53	3.64	5.04	3.90	4.14	3.71	3.17	0.51	2.37	3.13
8	2.05	4.60	2.47	3.94	7.09	1.64	2.98	3.51	1.71	2.99	3.16	-2.36	3.00	2.49
9	0.55	3.49	1.17	3.97	4.00	1.85	1.23	3.02	0.11	2.18	3.42	4.85	3.87	3.13
10	1.14	2.05	1.70	2.27	5.13	0.60	1.84	2.45	0.42	1.41	1.92	3.66	4.18	3.17
11	2.56	4.36	3.79	4.76	2.76	3.26	3.47	4.02	2.09	3.35	5.17	3.21	4.05	3.88
12	0.93	4.79	1.88	4.97	5.57	0.83	2.13	4.55	0.52	3.62	4.68	4.00	4.68	3.97
13	2.32	3.90	2.17	4.11	6.92	1.92	2.90	3.87	2.51	3.29	2.03	3.29	3.76	2.87
14	5.98	4.20	5.26	4.49	5.04	2.12	5.03	4.11	4.08	3.59	2.67	4.21	4.42	2.57
15	5.02	4.50	5.35	4.90	5.16	2.74	5.26	3.97	3.75	3.80	4.77	7.58	4.74	2.71
16	4.55	4.70	5.43	5.11	3.45	2.07	4.84	4.01	4.13	3.53	5.17	6.82	5.17	2.21
17	6.27	4.80	6.73	4.52	3.82	2.42	5.50	4.23	4.46	3.10	6.63	7.36	8.03	2.29
18	5.38	5.00	5.55	5.30	3.71	2.30	4.75	4.12	3.31	3.47	2.02	6.26	4.91	1.96
19	5.82	5.30	7.40	5.20	3.52	3.01	5.22	4.61	4.67	4.08	1.85	6.28	6.04	2.38
20	6.09	5.32	4.71	4.97	2.61	4.15	5.21	4.82	3.49	4.23	1.71	6.85	5.28	1.51
21	6.68		5.69		2.84		7.32		4.12		1.84	6.77	3.66	3.20
22	6.10		5.72		3.77		7.16		6.01		3.81	5.51	5.32	4.35
23	5.95		6.42		4.57		4.96		4.47		1.86	1.29	4.35	2.30
24	7.28		9.54		5.98		7.22		7.27		2.41		4.19	
25	1.55		2.08		9.46		2.59		1.88		1.97		4.15	
26	2.76		3.01		6.80		3.33		2.20		3.15		3.48	
27	3.06		3.27		5.08		3.15		2.83		3.68		4.69	
28	2.85		3.48		6.10		3.63		2.84		1.41		3.65	
29	6.27		6.17		2.85		5.82		4.72		3.47		4.06	
30	1.70		1.90		6.97		2.44		1.97		2.31		3.73	
31	4.10		5.18		2.57		4.96		3.92		1.51		3.03	
32	6.01		6.20		4.08		6.19		5.17		3.02		3.24	
33	3.12		2.88		3.22		6.11		8.04		3.02		3.97	
34	7.95		2.51		3.36		4.82		3.32		2.41		2.81	
35	3.37		3.34		2.73		3.69		3.18		0.23		3.33	
36	4.83		5.76		4.31		3.80		5.17		1.16		3.99	
37	3.82		4.04		4.61		4.49		3.95		1.30		3.53	
38	3.07		4.76		3.76		4.62		3.70		2.89		4.75	
39	1.73		1.85		8.87		2.77		2.41		3.16		4.98	
40	7.09		8.87		5.24		6.81		6.03		5.39		3.42	
41	4.87		5.85		4.97		5.90		4.52		2.42		5.01	
42	5.05		6.28		4.26		5.56		4.72		0.51		4.34	
43	6.07		6.53		6.25		6.28		6.08		1.92		4.70	
44	4.45		4.37		6.12		4.58		4.35		2.86		2.59	
45	5.14		5.25		3.03		5.21		4.22		3.65		2.70	
46	6.55		6.97		5.03		5.96		5.16		4.05		4.15	
47	2.78		3.58		4.71		4.22		2.70		2.28		3.42	
48	4.43		4.85		2.29		4.53		2.96		2.73		4.32	
49	6.09		6.72		3.76		5.36		4.84		0.62		4.14	
50	6.58		6.54		4.64		6.12		4.57		3.03		4.75	
51	5.53		6.21		4.07		4.96		3.41		11.29		4.98	
52	6.89		6.60		5.04		5.86		4.80		5.31		3.42	
53	4.04		4.92		4.22		5.14		3.51		6.01		5.01	
54	6.21		4.72		2.72		4.98		4.29		11.34		4.34	
55	4.57		5.41		5.19		5.82		3.80		5.61		4.70	
56	2.26		2.39		8.24		3.17		2.26		5.80		2.59	
57	6.02		6.97		3.03		5.21		4.35		2.01		3.03	
